# Genome-wide transcriptomic comparison of cotton (*Gossypium herbaceum*) leaf and root under drought stress

**DOI:** 10.1007/s13205-014-0257-2

**Published:** 2014-10-19

**Authors:** Alok Ranjan, Samir Sawant

**Affiliations:** 1 Department of Biotechnology, Faculty of Science, Banaras Hindu University, Varanasi, 221005 UP India; 2 National Botanical Research Institute, CSIR, Lucknow, 226001 UP India

**Keywords:** *G. herbaceum*, Drought tolerance, Leaf, Root, Pyrosequencing, Reactive oxygen species

## Abstract

In this study, the 454 pyrosequencing platform was used for analyzing the comparative transcriptomic profiles of leaf and root tissues of 1-month-old cotton (*Gossypium herbaceum*) plants under drought stress. A total of 56,354 and 49,308 reads were generated from leaf and root tissues, respectively, and clustered into 6,313 and 5,858 unigenes. The differentially expressed unigenes that showed up-regulation (≥2-fold) or down-regulation (2≤-fold) were considered for further analysis. A total of 3,517 unigenes were differentially expressed in both tissues. The 1,528 genes specific to leaves and 1,128 specific to roots were obtained. The 28 biological pathways in two tissues were found to respond significantly to drought stress. A total of 289 in leaf and 277 in root unknown (novel) unigenes were found to be remarkably regulated by drought stress. Some key regulatory genes involved in abiotic stress such as WRKY, ERF, AP2 EREBP, MYB, and LEA were highly expressed in leaves. The genes RHD3, LBD, and transcription factor WRKY75, known for root development under various stress conditions, were expressed specifically in root. The genes related to chlorophyll a/b binding protein and photosystem-related proteins showed significant higher expression in roots and as compared to leaves. It can be concluded that cotton leaves are distinct from roots in terms of molecular mechanisms for responses to drought stress.

**Electronic supplementary material:**

The online version of this article (doi:10.1007/s13205-014-0257-2) contains supplementary material, which is available to authorized users.

## Introduction

Drought is one of the major constraints to cotton production worldwide. Cotton plants grown under unsuitable environmental conditions such as temperature extremes, water deficit, and salinity stress face reduced growth and productivity resulting from loss of cotton boll and altered fiber development. Choice of *Gossypium herbaceum* in these tracts was not preferential but obligatory due to harsh cotton growing conditions. In most regions of India, *G. herbaceum* is planted for its natural textile fibre. To cope with water stress, plants execute a number of physiological and metabolic responses (Arndt et al. 2001; Dias et al. 2003). The physiological and biological functions of plants are determined by expression pattern of genes which are influenced by several adverse environmental conditions (Shinozaki and Yamaguchi-Shinozaki 2007; Valliyodan and Nguyen 2006). Hence, an importance in stress-related studies is to detect the expression of unique genes or clusters of genes and to determine their expression pattern in specific plant tissue or organs which control the regulatory network. The plant leaves provide an adaptive mechanism for plants in water-deficit condition by reducing the leaf area, decreasing transpiration rate, and maintaining stomatal closure (Caldeira et al. 2014; Sanchez-Blanco et al. 2009). The small leaves or reducing leaf expansion is an important adaptive mechanism for drought tolerance (Caldeira et al. 2014). The proportion of leaf elongation and size has been attributed to root variability, root length and growth (Parent et al. 2010). The root architecture is determined by an endogenous genetic program as well as by external environmental factors (Ishida et al. 2008; Smith and Smet 2012). The few studies that have compared leaf and root tissue transcriptomes have highlighted the organ specificity of drought responses (Cohen et al. 2010; Libault et al. 2010; Milner et al. 2014; Narsai et al. 2010). Roots sense the edaphic water deficit, send chemical signals to the shoots, and maintenance of root growth despite reduced water availability can contribute to drought tolerance through water foraging (Lynch 1995). The comparative analysis of gene expression profiles in the roots of maize (Jansen et al. 2013; Yue et al. 2008), soybean (Libault et al. 2010; You et al. 2011), populous (Cohen et al. 2010) and *Arabidopsis* has revealed a considerable number of root-specific genes and these are important for abiotic or biotic tolerance (Smith and Smet 2012). The various research groups using transcriptome studies have revealed several downstream components involved in the complex network of root formation (Ishida et al. 2008; Milner et al. 2014; Sengupta and Reddy 2011). A large number of genes encoding transcription factors (NLP, WRKY75, RAV and REM), osmoprotectants, ion transporters and heat shock proteins and pathways involved in hormone (ABA, ethylene and JA) biosynthesis and signal transduction are known to play an important role under drought stress (Narusaka et al. 2003; Ranjan et al. 2012b; Shinozaki and Yamaguchi-Shinozaki 2007; Tuteja and Sopory 2008). The genes involved in phenylpropanoid and flavonoid biosynthesis, pentose and glucuronate interconversions and starch and sucrose metabolism pathways are able to maintain adaptive condition during drought (Naika et al. 2013; Xiao et al. 2014). Cotton is an important source of natural fibre used in the textile industry and the productivity of the crop is adversely affected by drought stress (Trivedi et al. 2012; Zhu et al. 2013). Molecular processes in response to water-deficit stress have been studied at great length in cotton. Bowman et al. (2013) reported transcriptome analysis of upland cotton (*G. hirsutum*) root under drought stress. Chen et al. (2013) analyzed the drought-resistibility in two different cultivars of tetraploid upland cotton, using transcriptome profiling of leaf. Park et al. (2012) reported differentially expressed genes under water stress in root and leaf tissues in tetraploid cotton using cDNA-AFLP. Payton et al. (2011) investigated the transcriptional changes in the roots and leaves of diploid cotton under water-stress condition. The recent publication of the whole genome sequence of the cotton diploid relative *Gossypium raimondii* (Paterson et al. 2012) has expanded the use of NGS as a tool to study cotton development.

In our previous study we analyzed the drought-resistibility in different diploid cotton genotypes, and observed that the drought tolerance is due to several biochemical mechanisms working together (Ranjan et al. 2012a, b). In the present study, a genome-wide comparative analysis of genes in root and leaf tissue was performed to identify root and leaf specific genes and biological pathways that improve tolerance to drought stress. The comparative expression profiles of leaves and roots were useful in consolidating our knowledge of molecular mechanism of cotton pants in response to drought stress.

## Methods

### Drought stress treatment and plant growth

The genotypes of cotton (*G. herbaceum*), namely, RAHS-14 and GujCot-21 are drought sensitive and drought tolerant, respectively, were used for this study.

**Fig. 1:**
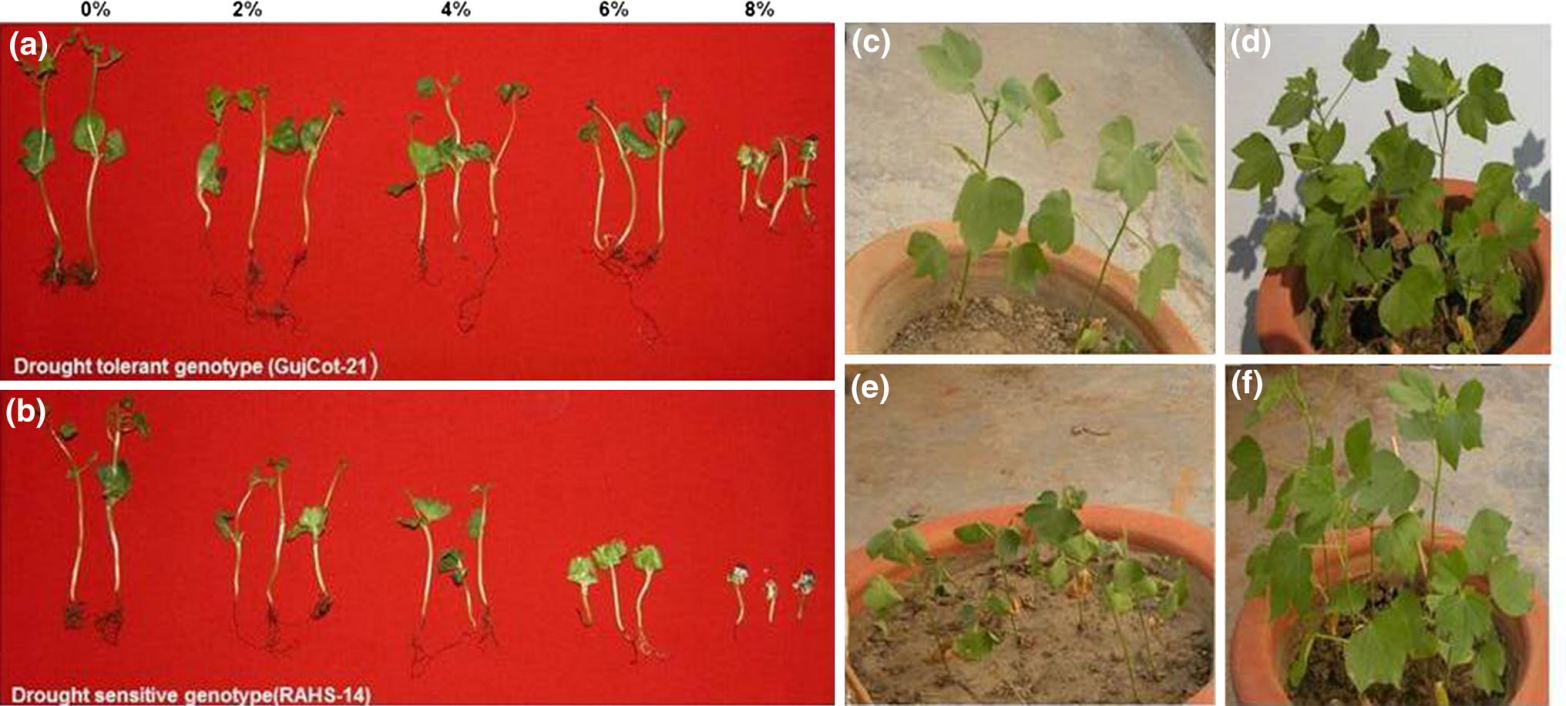
Morphological changes in plant leaf due to drought stress and variation in root length development due to osmotic stress in **a** GujCot-21 **b** RAHS-14. GujCot-21 grown under **c** drought stress condition, **d** control condition and, **e** RAHS-14 grown under drought stress condition and **f** control condition

The seeds of both genotypes were sterilized and aseptically kept immersed in water for a day at 30 °C and then placed for germination in a moist petri dish under the following conditions: 28/25 °C as day and night temperature, 12 h of light and dark periods alternatively and relative humidity of 80 % in the first experiment. After 3 days, properly germinated seeds were transferred on paper rafts, which were submerged (3/4th) in Hoagland’s media containing different percentages (2, 4, 6 and 8 %) of mannitol and mannitol free Hoagland’s media was considered as control (Fig. 1a, b). Then seedlings were allowed to grow until they had shown healthy growth in control condition then root lengths of grown seedlings were compared. In second experiment, plants were grown in the earthen pot, drought stress was given to the plants by withholding watering till soil moisture reaches below 30 % in pots and drooping effects on plant leaves became prominent (Fig. 1c, e), whereas the control pots were irrigated daily (Fig. 1d, f).

### RNA isolation

The earthen pot grown plants were selected for the experiments after drought stress treatment. The first leaves of RAHS-14 and root of GujCot-21 plant were collected for RNA extraction. Total RNA of leaf and root tissues was extracted by Spectrum Plant Total RNA Kit^®^ (Sigma-Aldrich) according to the manufacturer’s instructions. After DNase I treatment, RNA was purified by QIAquick PCR purification kit (Qiagen). Quality and quantity of the purified RNA were determined by measuring the absorbance at 260/280 nm (A260/A280). RNA integrity was further verified by 1.5 % agarose gel electrophoresis.

### Transcriptome sequencing

Total RNA (3 μg) from leaf and root tissue were reverse transcribed using a T7-Oligo (dT) Promoter Primer in the first-strand cDNA synthesis (Affymetrix). The double strand cDNA was synthesized and enriched using in vitro transcription (IVT) reaction (Affymetrix). cRNA (3 μg) was reverse transcribed in the first-strand cDNA synthesis step by using random hexamer primer, followed RNase H-mediated second strand cDNA synthesis and purified in column (QIAquick PCR purification kit) and further used for GS-FLX pyrosequencing. These sequence data were deposited in NCBI Sequence Read Archive (SRA, http://www.ncbi.nlm.nih.gov) with accession number SRX038499 (leaves) and SRX038496 (roots).

### Assembly of transcriptome using CAP3

The analyses of sequences were conducted using custom Perl scripts and publicly available software, R package (www.R.project.org). The sequences for leaves and roots were tagged with the library name and pooled exemplars were queried against the NCBI nucleotide database (NR) and in *Arabidopsis* TAIR-10 version using BLASTN at e-value 10^−10^ and alignment length of more than 50 % of the query sequence for annotation (see additional information-1, Table 1). All the *Gossypium* ESTs available at NCBI were downloaded and pooled. The pooled exemplars were also queried against all public cotton EST database to identify new transcripts of *Gossypium*.

**Table 1:** Assembly report of transcriptome sequencing data of *G. herbaceum* leaves and root under drought condition

Parameters	Leaf	Root
(a)		
Total reads	56,354	49,308
Total contigs (100 bp or greater)	6,313	5,858
Singleton	20,780	30,776
Average length of contigs (bp)	481.7 bp	532.9 bp
Supercontigs	101,063	104,298

	TAIR		Cotton EST		NR		No-hits
	Contigs	Singleton	Contigs	Singleton	Contigs	Singleton	Contigs
(b)							
Leaf	726	17,267	656	11,202	834	10,211	289
Root	682	16,806	488	12,383	713	9,885	275

(a) Total number of reads separated for both transcriptome libraries. Contigs generated by CAP3 assembly

(b) Contigs showing significant hits (e-value 10^−10^ and ≥50 % overlap) in the NCBI database. Contigs showing significant hits (e-value 10^−10^ and ≥50 % alignment of either the query or the subject) in the cotton EST database and TAIR-10

### Differential expression analyses

The reads of both the tissues were tagged and pooled to create a dataset of 105,662 reads and used for digital profiling. These reads were assembled using the CAP3 programme at an overlap of 100 bp and 80 % identity. These reads assembled into 12,171 contigs which include only those contigs that have more than five reads. We calculated TPM value and* R* value using the R statistics for contigs and those with* R* value ≥3 and fold change ≥2 were considered significantly differentially expressed contigs. These filtered contigs were annotated using BLASTN against NCBI nucleotide (NT) database and BLASTX against NCBI non-redundant proteins (NR) (see additional information-2).

### Heat map analysis

For visualization of the significant comparisons, heat maps of the significant genes after FDR adjustment were produced with the heat maps two package in R. Hierarchical clustering of individual sample with 1,000 bootstrap replications was performed with the R package pvclust and heat map were sorted accordingly. To visualize clusters of genes expression, we grouped the z-transformed expression ratios by using k-means in R.

### GO analyses

The GO annotations for the sequences were derived using their UniProt annotation. The UniProt database was used as it has extensive GO mapping. The GO annotation for level 5 was extracted for each library and used for further analysis.

### Biochemical pathway analysis

Pathway analysis was performed to find out the significant pathways of the differentially expressed genes in roots and leaves according to the KEGG databases. The two-way Fisher’s exact test and* χ* ^2^-test were used to classify the significant pathways, and the threshold of significance was defined by *p* < 0.05 and FDR <0.05. The FDR value was used to correct the ‘*p* value’ (Garcia-Alcalde et al. 2011).

### MapMan analysis

To adapt MapMan software to cotton (*G. herbaceum*) the major MapMan BINs and their sub-BINS used for the classification of *Arabidopsis* genes were transferred to the *G. herbaceum* system. The classification based on the similarity to *Arabidopsis* proteins was then automatically checked against domains that have been assigned to a MapMan category (Usadel et al. 2006). Furthermore, cotton tentative consensus sequences that did not meet the prerequisites for an automatic draft assignment were classified manually according to their respective TIGR annotation (TIGR release 10). Finally, all draft assignments were corrected manually for potential mistakes.

## Results

### Comparison of drought tolerance of GujCot-21 and RAHS-14

The GujCot-21 and RAHS-14 exhibited contrasting difference in their root growth under osmotic stress and control condition. GujCot-21 has longer root length as compared to RAHS-14 at 6 % mannitol concentration (Fig. 1a, b). In earthen pot experiments, RAHS-14 showed prominent effect of drought stress (Fig. 1e) as compared to control (Fig. 1d). However, GujCot-21 showed much better development, less wilting and higher biomass as compared to RAHS-14 (Fig. 1c). In addition, GujCot-21 exhibited better drought tolerance than RAHS-14 as observed by visual comparison of leaf senescence and wilting symptom in two genotypes (Fig. 1c, f.)

### Overall features of the drought stress-responsive expression profiles in leaves and roots

In leaf tissue 1,528 genes were identified; out of these 289 genes showed no hits to any protein or nucleotide in the database (NR-non redundant and NT-nucleotide data base of NCBI) and the 26 genes were hypothetical or unknown proteins were obtained (see additional information 2). The majority of the annotated genes were from the photosynthesis pathway, with high expression in ribulose 1–5 bisphosphate carboxylase/oxygenase activase, photosystem II D and chlorophyll a/b binding proteins. The senescence-associated proteins were also observed in the differentially expressed genes. In root library 1,128 genes were identified and out of these 275 genes did not have any hit to any of the database (NR, UniProt, NT) and about 8 % genes matched with predicted/hypothetical proteins. The other genes related to drought stress that were upregulated in the root were ascorbate peroxidase, cysteine protease, delta-tonoplast intrinsic proteins, Lea proteins, etc. (see additional information 2).

### Roots behave in a more focused response under drought stress compared to leaves

**Fig. 2:**
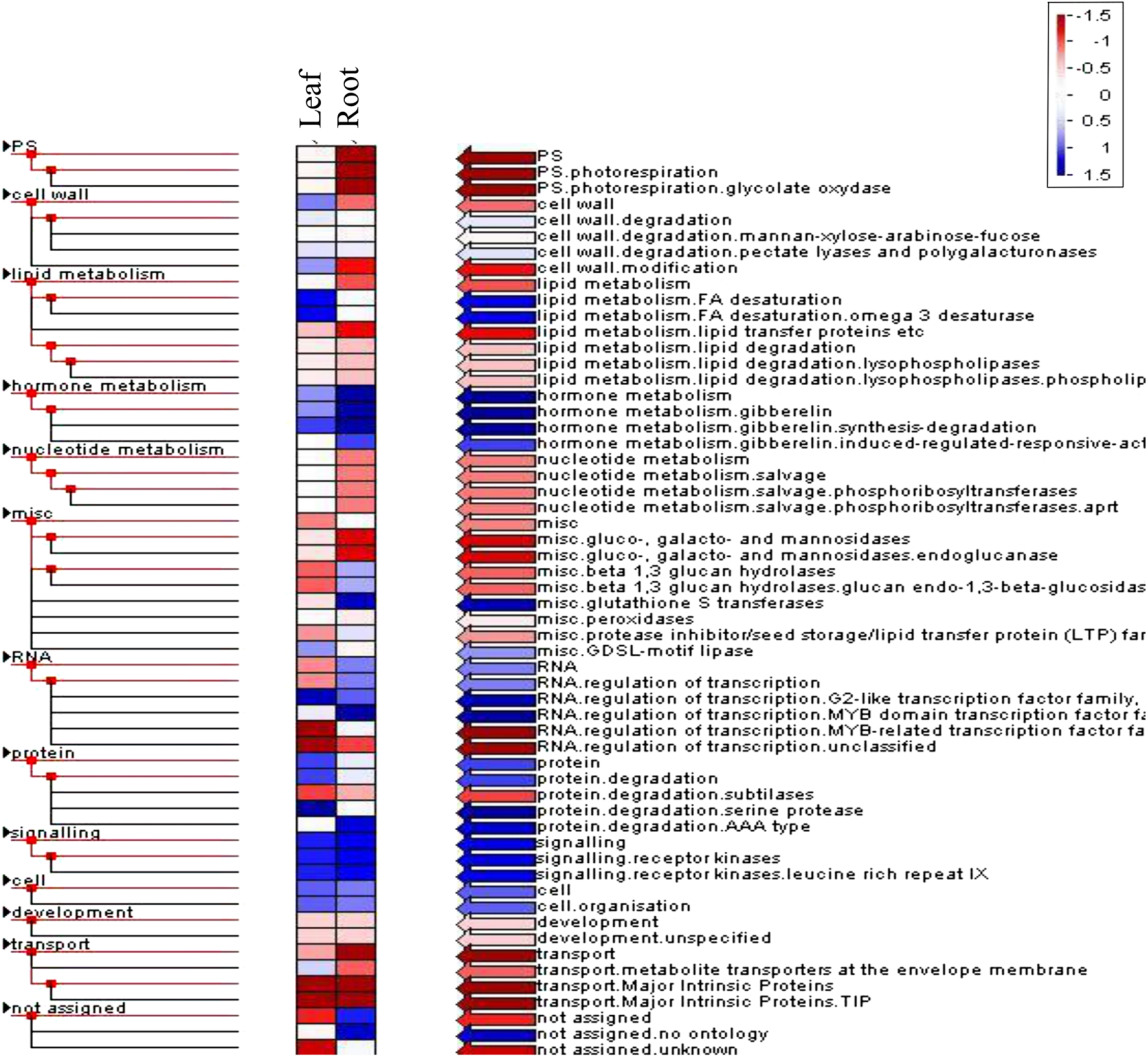
MapMan analysis of differentially expressed genes in leaf and root

**Fig. 3:**
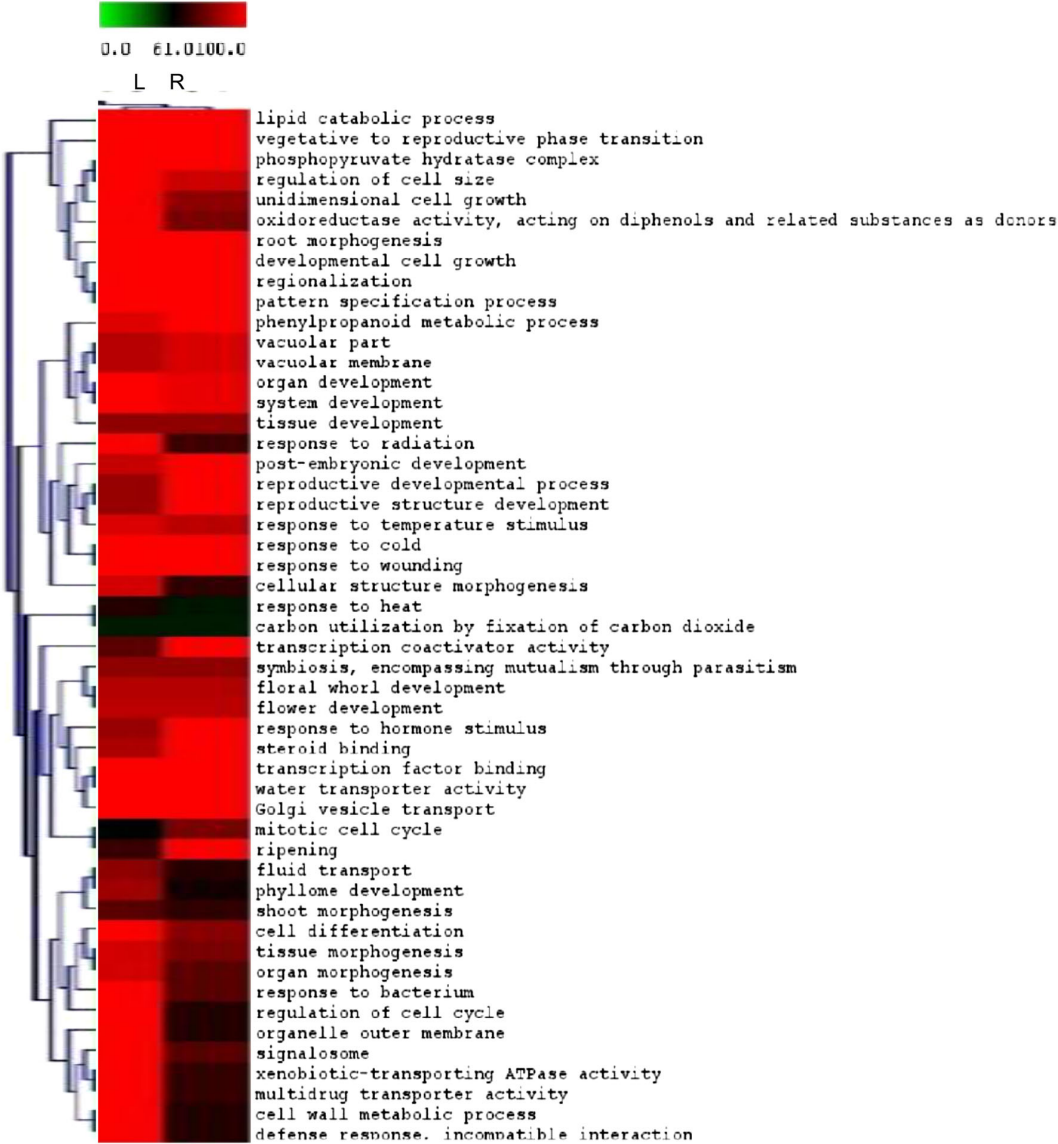
Heat maps of expression profiles of top ranked differentially expressed genes in leaf and root

Among the cotton genes, 6,313 and 5,858 from leaves and roots, respectively, were either twofold up- or down-regulated with adjusted *p* ≤ 0.05. Together, 12,171 genes were either significantly up- or down-regulated under drought stress (Table 1). Thus 3,588 genes were differentially regulated in both leaves (1,528) and roots (1,128). However, various tissues behaved differently during the stress response. In terms of the number of genes, there appeared to be a balance between leaves and roots, while in terms of induction high number of genes was primarily observed in the root (see additional information 2). To have a general view of the whole picture, we grouped and classified them into Gene Ontology terms then biological processes were further examined. There were five significantly represented GOs in leaves while roots showed eight indicating the earlier response of roots. It is likely because of the faster dehydration on root represented by shorter root length (Table 2; Fig. 1e); in other words, roots are more water stressed than leaves. In the field, root also is the earlier organ under drought because plants uptake water through their roots. When soils become dry, roots sense this water deficit and produce chemical signals to transport to leaves via the xylems resulting in physiological changes in leaves (Arndt et al. 2001; Sengupta and Reddy 2011; Tuteja and Sopory 2008). Further we analyzed these genes by MapMan, This finding suggested that, the leaves differentially express genes in a more random/scattered manner that lead to less statically significant results. Compared to that, roots seemed to behave in a more focused response. Unlike the responses of the leaves, root tissue showed notable expression of stress related and protein signaling pathway (Fig. 2). The results suggest that, under drought stress the leaves try to maintain normal functional condition and re-direct to the whole system on stress response, whereas, root tissues became more active. The heat map analysis showed that the photosynthesis and related processes were among enhanced processes in the roots, while it was repressed in leaves, In particular, the simultaneous reduction in leaves and induction in root of photosynthesis as well as secondary metabolism suggests the possibility of biological processes functioning in an opposite and tissue-specific manner (Fig. 3).

**Table 2:** Commonly regulated GO terms in leaf and root

Differentially regulated GO terms in leaf and root			*p* value
GO annotation	GO terms	Leaf	Root
GO:0006950	Response to stress	0.0006	0.0058
GO:0009725	Response to hormone stimulus	0.0002	0.0049
GO:0009644	Response to high light intensity	0.0021	0.0009
GO:0009408	Response to heat	0.0047	0.0012
GO:0009266	Response to temperature stimulus	0	0
GO:0009808	Lignin metabolic process	0	0.0017
GO:0042221	Response to chemical stimulus	0	0
GO:0006720	Isoprenoid metabolic process	0.029	0.0341
GO:0009719	Response to endogenous stimulus	0.0358	0.0296
GO:0009725	Response to hormone stimulus	0.0361	0.0122
GO:0008610	Response to lipid metabolism	0.0359	0.00165
GO:0009628	Response to abiotic stimulus	0.0053	0.002
GO:0050896	Response to stimulus	0.0017	0.0014
GO:0051716	Cellular response to stimulus	0.0021	0.0032
GO:0032268	Regulation of cellular protein metabolic process	0.0029	0.0065
GO:0009314	Response to radiation	0.0024	0.0067
GO:0042221	Response to chemical stimulus	0.00032	0.0009
GO:0042254	Ribosome biogenesis	0.004	0.001
GO:0050896	Response to stimulus	0	0.008
GO:0034470	ncRNA processing	0	0.002
GO:0043412	Biopolymer modification	0	0
GO:0043687	Post-translational protein modification	0	0
GO:0006464	Protein modification process	0.0138	0.002
GO:0006793	Phosphorus metabolic process	0	0
GO:0006796	Phosphate metabolic process	0.0165	0.0114
GO:0008152	Metabolic process	0.0113	0
GO:0016072	rRNA metabolic process	0.0117	0
GO:0006261	DNA-dependent DNA replication	0.0022	0
GO:0006364	rRNA processing	0.013	0.0008
GO:0006952	Defense response	0	0
GO:0051704	Multi-organism process	0.0055	0
GO:0007166	Cell surface receptor linked signal transduction	0.0054	0
GO:0008283	Cell proliferation	0.0023	0.0062

### Differentially regulated cellular metabolism in leaves and roots

To identify the stress affected biochemical network of cells, we retrieved *Arabidopsis* homologs of cotton genes from PLEXdb (http://www.plexdb.org/) and calculated the effected pathway using KOBAS. Table 3 shows the 28 biological pathways were commonly affected in roots and leaves and the majority of affected pathways were observed in leaves. Some pathways were significantly affected in both leaves and roots; they’re involved in energy production, biosynthesis of phytohormones, amino acids, pigments and many others. Not only did each tissue differently regulate genes within the shared pathway, they also possessed tissue specific reactions. Specifically, leaves showed strong induction of choline and trehalose biosyntheses when cellulose and initial step of fatty acid biosynthesis were negatively regulated only in root (Table 3).

**Table 3:** Different biochemical pathways significantly affected under drought stress in leaf and root tissue

Biological pathways	*p* value	
	Leaf	Root
Beta alanine metabolism	0.022	0.033
Histidine metabolism	0.043	0.026
Terpenoid backbone biosynthesis	0.026	0.017
Fatty acid metabolism	0.041	0.027
Flavonoid biosynthesis	0.042	0.024
Glycolysis/gluconeogenesis	0.036	0.034
Spliceosomes	0.0003	0.048
Ubiquitin mediated proteolysis	0.029	0.0036
Mevalonate pathways	0.028	0.00001
Tryptophan biosynthesis	0.044	8.63E-06
Carotenoid biosynthesis	6.35E-06	0.000045
Starch degradation	0.026	0.00137
IAA biosynthesis	0.046	0.000039
Ascorbate glutathione cycle	0.018	0.0019
Carotenoid biosynthesis	0.023	0.024
Sterol biosynthesis	0.048	0.034
Glycine degradation	0.030	0.041
Cysteine biosynthesis	0.024	0.040
Triacylglycerol degradation	0.007	0.041
Chlorophyll biosynthesis	0.043	0.033
Lignin biosynthesis	0.033	0.036
Choline biosynthesis	0.011	0.014
Trehalose biosynthesis	0.022	0.008
Asparagine degradation I	0.024	0.046
Calvin cycle	0.00042	6.02E-07
Fatty acid elongation saturated	0.041	1.75E-06
Threonine degradation	0.0003	0.00012
Cellulose biosynthesis	0.024	2.43E-07

### Unique and shared transcription factors in roots and leaves in response to drought stress

TFs are the main components to understand the complexity of expression of stress induced genes in their signaling network as suggested in various studies (Agarwal et al. 2006a, b; Ranjan et al. 2012b). In order to further investigate on these molecules, we determined the number of unique and shared TF’s expressed in leaves and roots using conserved domain database in NCBI. Among 29 groups that are affected by drought, three and six were uniquely expressed in leaves and roots, respectively. MybDNA-binding bHLH, HSF and WRKY were the most strongly affected groups even though the expressions were different in both the tissue. Transcription factors, such as WRKY 75, RAV, REM, CAMTA, SBP, NLP and G2 like, Orphan, TCP were uniquely expressed in root and leaf tissue, respectively (Table 4).

**Table 4:** Differentially and uniquely expressed transcription factors in leaf and root under drought

TFs	Differentially expressed		Uniquely expressed	
	Leaf	Root	Leaf	Root
ARF	9	4	–	–
bHLH	16	11	–	–
bZIP	3	5	–	–
BZR	6	2	–	–
C2C2-like	7	9	–	–
C2C2-YABBY	3	6	–	–
C2C2	1	7	–	–
C3H	5	4	–	–
CCAAT-HAP5	6	8	–	–
EIL	2	2	–	–
G2-like	–	–	1	–
GRAS	6	4	–	–
GRF	8	4	–	–
Homeobox	3	7	–	–
HSF	11	8	–	–
MAKS	7	4		
MYB	19	13		
NAC	5	2		
NLP	–	–	–	4
Orphan	–	–	2	–
SBP	–	–	–	3
TCP	4	5	–	–
WRKY	12	9	–	–
CAMTA	–	–	–	7
REM	–	–	–	6
TCP	–	–	8	–
RAV	–	–	–	4
WRKY75	–	–	–	8

### Drought enhanced the expression of photosynthesis gene in root

**Fig. 4:**
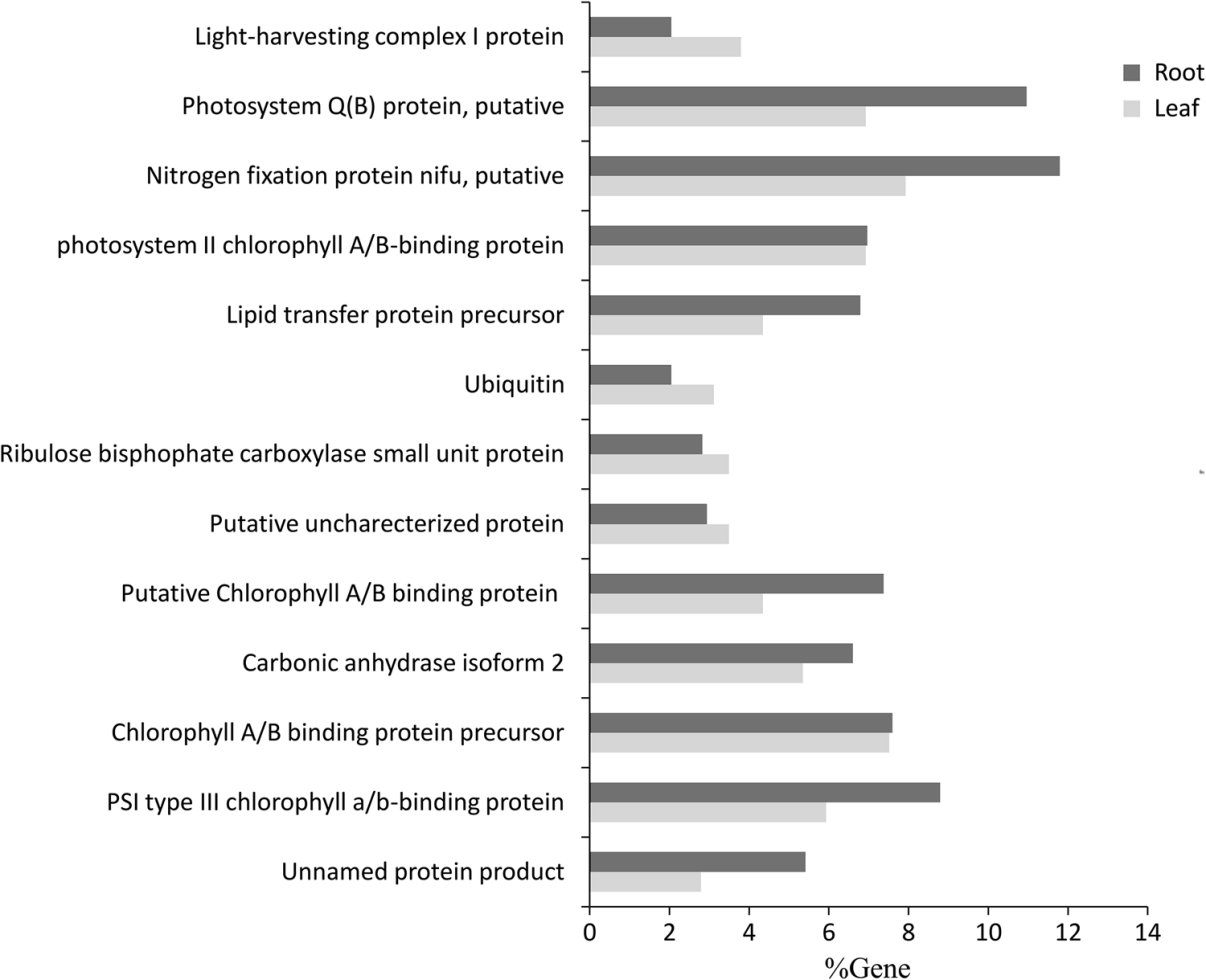
Graphical comparison of photosynthesis related genes in leaf and root

In general the photosynthesis related genes are expressed at a lower level in roots as compared to the expression in leaves. However, in this study the expression pattern is very different. Photosynthesis genes that are encoded separately in plastid and nuclear genome are responsible for photosynthesis core subunits, and light harvesting complex proteins and pigment biosynthesis; respectively (Gutierrez-Nava et al. 2004; Kino-oka et al. 2001). The genes related to chlorophyll a/b binding protein and photosystem-related proteins showed significant higher expression in roots and as compared to leaves (Fig. 4). It is well known that light and plastid development are both crucial for the expression of nuclear photosynthesis genes. Although in our analysis no biological process or biological pathways shows plastid related biosynthesis in root, we found that biological pathway related to “ribulose-1,5-bisphosphate carboxylase” (RuBisCO) was significantly high in leaves.

### Defense genes dominantly expressed in roots as compared to leaves

Through long-term evolution and adaptation to extreme conditions, cotton has been found to be rich in resistance genes for abiotic stresses (Wendel and Cronn 2003). When cotton plants face water stress, due to the change in external environment, plants are able to activate a series of mechanisms to respond drought stress (Park et al. 2012; Ranjan et al. 2012a, b). Cells recognize external stresses and induce many signal proteins, including signaling receptor kinases, calcium-dependent protein kinases and G-proteins. In our results showed that the transcripts of these signal proteins accumulated in both tissues during drought stress. Genes for peroxidase, glutathione peroxidase, redox metabolism and superoxide dismutase, were predominantly expressed in root tissue (Table 5). These genes are involved in scavenging reactive oxygen species (ROS), and play a pivotal role as triggers of gene expression during abiotic stresses (Miller et al. 2010; Ranjan et al. 2012b).

**Table 5:** Differentially expressed antioxidant genes in leaf and root tissues

Contigs represent antioxidant genes	Contigs represent number of expressed contigs		Description
	Root	Leaf	
Contig00283	305	0	Peroxidase
Contig00097	74	60	Peroxisomal enoyl-CoA hydratase
Contig00254	7	9	Peroxiredoxin
Contig00468	9	20	Cytoplasmic Cu/ZnSOD
Contig00520	5	16	Glutamate synthase
Contig00525	11	1	Metallothionein-like type 1 protein
Contig00718	1	21	Cu/Zn superoxide dismutase
Contig00763	2	9	Peroxiredoxin
Contig00867	8	2	Glutathione S-transferase
Contig01025	10	5	Glutathione S-transferase
Contig01228	8	0	Peroxidase 17
Contig01314	20	7	Superoxide dismutase
Contig01348	22	0	Superoxide dismutase
Contig01685	26	0	Glutathione reductase
Contig01694	6	0	Sulfite oxidase
Contig01756	5	1	Thioredoxin H7
Contig01852	8	3	Catalase
Contig01916	2	4	Peroxisomal targeting signal type 1
Contig02183	3	3	Ascorbate peroxidase
Contig02352	9	9	Ascorbate peroxidase
Contig02464	1	2	Glutathione S-transferase
Contig02486	2	0	Glutathione S-transferase U1
Contig02488	13	1	Ascorbate peroxidase
Contig02585	1	1	Lipoxygenase
Contig00114	95	0	Superoxide dismutase
Contig00697	26	0	Glutathione peroxidase
Contig03151	17	0	Glutathione peroxidase
Contig02529	9	0	Catalase

## Discussion

The advent of pyrosequencing has enabled to obtain the transcriptional changes of entire genome of a particular plant or specific plant organs in response to various stresses. However, the whole genome sequences are still not available for *G. herbaceum* species of cotton, leading to a dependence on mRNA sequences by pyrosequencing or collections of ESTs assembled from random cDNA libraries. In this study, we used the 454 pyrosequencing platform for transcriptome sequencing of cotton (*G. herbaceum*) leaf and root tissues. It was observed that GujCot-21 and RAHS-14 showed substantial differences in root growth under osmotic stress (Fig. 1a, b) and leaf senescence under drought stress condition (Fig. 1c, f). The leaf and root both tissues shared some common gene expression during stress, with the purpose of enhancing protective systems. However, these two tissues appeared to respond differently to drought conditions. Leaves induced more genes than root but those genes were scattered in many processes, most significantly were productions of osmoprotectant and senescence associated genes. The genes expressed in roots mainly associated with antioxidant and photosynthesis related pathways (Table 5; Fig. 4). Chen et al. (2013) recently reported down regulated expression of chloroplast-/plastid-related genes in leaf tissue of drought tolerant cultivar of cotton as compare to drought sensitive cultivar, while Bowman et al. (2013), observed higher expression of genes in root associated with starch and sugar metabolism is similar to our results. Besides, the two tissues can affect each other via the signaling and transportation system. Through long-term selection and adaptation to extreme conditions, cotton has been found to be rich in resistance genes for a range of abiotic stresses, including drought, high temperature and salinity (Ranjan et al. 2012a; Zhou et al. 2014). Chen et al. (2013) results also suggested that high number of genes are expressed temporally and specifically in plant response to drought treatment.

Transcription regulation plays a central role in stress signal transduction pathways. In this study, we found 29 group of transcription factor genes differentially regulated at different levels in roots and leaves in response to drought stress. Among these, TF genes some were unique to leaf and root tissues. This finding reveals that a large group of TF genes are involved in the transcription regulation in response to drought stress in leaves and roots in a specific manner when cotton plant face drought stress. Our study also showed that a large proportion (289 in leaves and 278 in roots) of expressed unigenes have no functional annotation or have unknown function. It is also noteworthy that many of these unigenes showed remarkable expression changes in response to drought stress, especially with some having a unique expression in leaf and root tissues. Our results confirm differential expression of transcription factors in response to water-deficit stress, as has been observed in many plant species, including cotton (Park et al. 2012, Bowman et al. 2013). Therefore, it is possible to identify some drought responsive genes unique to cotton. Further analysis of the functions and expression controlling mechanism of these genes in cotton would not only supply the opportunity of isolation and identification of novel genes, but also enhance our further understanding of specific mechanism of drought tolerance in cotton.

## Conclusions

To date, a limited number of studies on drought stress mediated gene expression in cotton have been reported. In this study we described an analysis of gene expression in cotton leaves and roots in response to drought stress and intended to carry out a comparative transcript profiling between the leaves and roots. We focused on a set of transcripts that exhibited unique expression in leaves and roots of cotton under drought stress. This is the first trial to comprehensively explore the genome-wide gene expression patterns in leaf and root of drought responsiveness in cotton. Our results show that cotton leaves are distinct from roots in terms of molecular mechanisms for responses to drought stress.

## Electronic supplementary material

Below is the link to the electronic supplementary material.

Supplementary material 1 (XLSX 1935 kb). Additional information-1 Blast detail of leaf and root contigs

Supplementary material 2 (XLSX 637 kb). Additional information-2 Uniquely and differentially expressed genes in leaf and root tissue

## References

[CR1] Agarwal M, Hao Y, Kapoor A, Dong CH, Fujii H, Zheng X, Zhu JK (2006). A R2R3 type MYB transcription factor is involved in the cold regulation of CBF genes and in acquired freezing tolerance. J Biol Chem.

[CR2] Agarwal PK, Agarwal P, Reddy MK, Sopory SK (2006). Role of DREB transcription factors in abiotic and biotic stress tolerance in plants. Plant Cell Rep.

[CR3] Arndt SK, Clifford SC, Wanek W, Jones HG, Popp M (2001). Physiological and morphological adaptations of the fruit tree Ziziphus rotundifolia in response to progressive drought stress. Tree Physiol.

[CR100] Bowman MJ, Park W, Bauer PJ, Udall JA, Page JT et al (2013) RNA-seq transcriptome profiling of upland cotton (*Gossypium hirsutum* L.) root tissue under water-deficit stress. PLoS ONE 8(12):e82634 10.1371/journal.pone.0082634 PMC3855774 24324815

[CR4] Caldeira CF, Bosio M, Parent B, Jeanguenin L, Chaumont F, Tardieu F (2014) A hydraulic model is compatible with rapid changes in leaf elongation rate under fluctuating evaporative demand and soil water status. Plant Physiol 164:1718–1730 10.1104/pp.113.228379 PMC3982736 24420931

[CR101] Chen Y, Liu Z-H, Feng L, Zheng Y, Li D-D et al (2013) Genome-wide functional analysis of cotton (*Gossypium hirsutum*) in response to drought. PLoS ONE 8(11):e80879 10.1371/journal.pone.0080879 PMC3832458 24260499

[CR5] Cohen D, Bogeat-Triboulot MB, Tisserant E, Balzergue S, Martin-Magniette ML, Lelandais G, Ningre N, Renou JP, Tamby JP, Le Thiec D, Hummel I (2010). Comparative transcriptomics of drought responses in Populus: a meta-analysis of genome-wide expression profiling in mature leaves and root apices across two genotypes. BMC Genom.

[CR6] Dias AP, Brown J, Bonello P, Grotewold E (2003). Metabolite profiling as a functional genomics tool. Methods Mol Biol.

[CR7] Garcia-Alcalde F, Garcia-Lopez F, Dopazo J, Conesa A (2011). Paintomics: a web based tool for the joint visualization of transcriptomics and metabolomics data. Bioinformatics.

[CR8] Gutierrez-Nava Mde L, Gillmor CS, Jimenez LF, Guevara-Garcia A, Leon P (2004). Chloroplast biogenesis genes act cell and noncell autonomously in early chloroplast development. Plant Physiol.

[CR9] Ishida T, Kurata T, Okada K, Wada T (2008). A genetic regulatory network in the development of trichomes and root hairs. Ann Rev Plant Biol.

[CR10] Jansen L, Hollunder J, Roberts I, Forestan C, Fonteyne P, Van Quickenborne C, Zhen RG, McKersie B, Parizot B, Beeckman T (2013). Comparative transcriptomics as a tool for the identification of root branching genes in maize. Plant Biotechnol J.

[CR11] Kino-oka M, Nagatome H, Taya M (2001). Characterization and application of plant hairy roots endowed with photosynthetic functions. Adv Biochem Eng Biotechnol.

[CR12] Libault M, Farmer A, Brechenmacher L, Drnevich J, Langley RJ, Bilgin DD, Radwan O, Neece DJ, Clough SJ, May GD, Stacey G (2010). Complete transcriptome of the soybean root hair cell, a single-cell model, and its alteration in response to Bradyrhizobium japonicum infection. Plant Physiol.

[CR13] Lynch J (1995). Root architecture and plant productivity. Plant Physiol.

[CR14] Miller G, Suzuki N, Ciftci-Yilmaz S, Mittler R (2010). Reactive oxygen species homeostasis and signalling during drought and salinity stresses. Plant Cell Environ.

[CR15] Milner MJ, Mitani-Ueno N, Yamaji N, Yokosho K, Craft E, Fei Z, Ebbs S, Zambrano MC, Ma JF, Kochian LV (2014) Root and shoot transcriptome analysis of two ecotypes of Noccaea caerulescens uncovers the role of NcNramp1 in Cd hyperaccumulation. Plant J 78:398–410 10.1111/tpj.12480 24547775

[CR16] Naika M, Shameer K, Sowdhamini R (2013). Comparative analyses of stress-responsive genes in Arabidopsis thaliana: insight from genomic data mining, functional enrichment, pathway analysis and phenomics. Mol BioSyst.

[CR17] Narsai R, Castleden I, Whelan J (2010). Common and distinct organ and stress responsive transcriptomic patterns in Oryza sativa and Arabidopsis thaliana. BMC Plant Biol.

[CR18] Narusaka Y, Nakashima K, Shinwari ZK, Sakuma Y, Furihata T, Abe H, Narusaka M, Shinozaki K, Yamaguchi-Shinozaki K (2003). Interaction between two cis-acting elements, ABRE and DRE, in ABA-dependent expression of arabidopsis rd29A gene in response to dehydration and high-salinity stresses. Plant J.

[CR19] Parent B, Suard B, Serraj R, Tardieu F (2010). Rice leaf growth and water potential are resilient to evaporative demand and soil water deficit once the effects of root system are neutralized. Plant Cell Environ.

[CR20] Park W, Scheffler BE, Bauer PJ, Campbell BT (2012). Genome-wide identification of differentially expressed genes under water deficit stress in upland cotton (*Gossypium hirsutum* L.). BMC Plant Biol.

[CR21] Paterson AH, Wendel JF, Gundlach H, Guo H, Jenkins J (2012). Repeated polyploidization of gossypium genomes and the evolution of spinnable cotton fibres. Nature.

[CR22] Payton P, Kottapalli KR, Kebede H, Mahan JR, Wright RJ, Allen RD (2011). Examining the drought stress transcriptome in cotton leaf and root tissue. Biotechnol Lett.

[CR23] Ranjan A, Nigam D, Asif MH, Singh R, Ranjan S, Mantri S, Pandey N, Trivedi I, Rai KM, Jena SN, Koul B, Tuli R, Pathre UV, Sawant SV (2012). Genome wide expression profiling of two accession of G. herbaceum L. in response to drought. BMC Genom.

[CR24] Ranjan A, Pandey N, Lakhwani D, Dubey NK, Pathre UV, Sawant SV (2012). Comparative transcriptomic analysis of roots of contrasting gossypium herbaceum genotypes revealing adaptation to drought. BMC Genom.

[CR25] Sanchez-Blanco MJ, Alvarez S, Navarro A, Banon S (2009). Changes in leaf water relations, gas exchange, growth and flowering quality in potted geranium plants irrigated with different water regimes. J Plant Physiol.

[CR26] Sengupta D, Reddy AR (2011). Water deficit as a regulatory switch for legume root responses. Plant Signal Behav.

[CR27] Shinozaki K, Yamaguchi-Shinozaki K (2007). Gene networks involved in drought stress response and tolerance. J Exp Bot.

[CR28] Smith S, De Smet I (2012). Root system architecture: insights from arabidopsis and cereal crops. Philos Trans R Soc Lond B Biol Sci.

[CR29] Trivedi I, Ranjan A, Sharma YK, Sawant S (2012). The histone H1 variant accumulates in response to water stress in the drought tolerant genotype of Gossypium herbaceum L. Protein J.

[CR30] Tuteja N, Sopory SK (2008). Chemical signaling under abiotic stress environment in plants. Plant Signal Behav.

[CR31] Usadel B, Nagel A, Steinhauser D, Gibon Y, Blasing OE, Redestig H, Sreenivasulu N, Krall L, Hannah MA, Poree F, Fernie AR, Stitt M (2006). Pageman: an interactive ontology tool to generate, display, and annotate overview graphs for profiling experiments. BMC Bioinform.

[CR32] Valliyodan B, Nguyen HT (2006). Understanding regulatory networks and engineering for enhanced drought tolerance in plants. Curr Opin Plant Biol.

[CR33] Wendel JF, Cronn RC (2003). Polyploidy and the evolutionary history of cotton. Adv Agron.

[CR34] Xiao X, Tang C, Fang Y, Yang M, Zhou B, Qi J, Zhang Y (2014). Structure and expression profile of the sucrose synthase gene family in the rubber tree: indicative of roles in stress response and sucrose utilization in the laticifers. FEBS J.

[CR35] You J, Zhang H, Liu N, Gao L, Kong L, Yang Z (2011). Transcriptomic responses to aluminum stress in soybean roots. Genome.

[CR36] Yue G, Zhuang Y, Li Z, Sun L, Zhang J (2008). Differential gene expression analysis of maize leaf at heading stage in response to water-deficit stress. Biosci Rep.

[CR37] Zhou M, Sun G, Sun Z, Tang Y, Wu Y (2014) Cotton proteomics for deciphering the mechanism of environment stress response and fiber development. J Proteomics 105:74–84 10.1016/j.jprot.2014.03.017 24680693

[CR38] Zhu YN, Shi DQ, Ruan MB, Zhang LL, Meng ZH, Liu J, Yang WC (2013). Transcriptome analysis reveals crosstalk of responsive genes to multiple abiotic stresses in cotton (*Gossypium hirsutum* L.). PLoS ONE.

